# St. Thomas's Hospital

**Published:** 1916-09-30

**Authors:** 


### NATIONAL INSURANCE AND THE VOLUNTARY HOSPITALS.

### St. Thomas's Hospital.

WHEN the National Insurance Acts first came into operation the treasurer and governors of St. Thomas's Hospital made a definite rule that insured persons should not be treated unless they required treatment other than could be afforded by a general practitioner of ordinary professional competence and skill. The decision, therefore, as to which insured persons need hospital treatment is based in the first instance on the medical examination which is given to all patients, insured and uninsured alike. The patients then go to one of the lady almoner's offices, where all insured patients whose hospital letter has not been marked by the doctor as requiring special treatment are dismissed (see table below). A certain number of cases are held over daily, pending the result of tests in the clinical laboratory, or until a skiagram has been taken. The results of the tests and skiagrams are then communicated to the panel doctors. In addition to this, a very large number of panel patients bring with them letters from their panel doctors, which are answered by the medical staff, the patients' letters being marked "Opinion only," and no treatment is given. In every instance, when dismissing a patient after the medical examination, the lady almoner explains clearly that the hospital is open to them in the future should they require hospital treatment, and that their doctor is at liberty to write to the hospital if he wants an opinion or suggestion as to the present treatment required, or to come up to the hospital to consult on the case. Before the war the co-operation between the hospital and the panel doctors was becoming very apparent, but of necessity the pressure on all forms of medical service has temporarily curtailed it.

Thus, while definitely refusing to accept insured out-patients who can be treated by their panel doctors, the governors of the hospital have done all in their power to assist the panel doctors, and give them the benefit of hospital diagnosis and examination. It must be remembered that the National Insurance Acts (except in the case of tuberculosis) have not provided for *all* the medical treatment required by insured patients, but only for the treatment of minor ailments; the panel doctors are daily referring patients to the hospital, and, therefore, it would seem that in the future the out-patients' department will tend more and more to develop into a consultative centre with a large number of special departments for treatment. In one respect the State scheme would appear to be a retrograde one. Of late years the medical staff have depended more and more on the social service department of the hospital to supervise the patients' homes, to instruct them in hygiene, and to provide the suitable nourishment, temporary allowances, and special diet that they require. But the panel doctor has no medico-social worker beside him, and the habit of drug-taking as a cure for illness again holds sway. With the best intentions it is absolutely impossible for a busy panel doctor to give instructions in hygiene or to supervise his patients' home lives. The factory girl who is sleeping in an unhealthy room with the windows hermetically sealed, and subsisting on a diet of fried fish, pickles, and strong tea, continues to do so fortified by the bottle of medicine, and when at last she finds she is getting worse, she applies at the hospital, saying that the panel medicine was not strong enough. Again, the panel doctor tells the workman that he needs artificial teeth, or a month at the sea, or special diet, but has no clearing house to which he can refer such patients, no almoner who is trained to carry out such directions, so the patient disregards the instructions, but carries off the bottle of medicine which may soothe the symptoms, but cannot touch the root of the trouble. It would therefore seem that, sooner or later, unless the working classes are to be relegated again to the tyranny of drugs, the State will have to appoint some centre for social service to each group of panel doctors, to which patients can be referred for treatment, and which will be in close communication with each doctor.

The following table gives statistics of the number of insured patients who attended the casualty and out-patient departments of St. Thomas's Hospital since the Insurance Acts came into force, and the proportion considered in need of hospital treatment.

| — | Total Number of Insured Patients who presented themselves for Treatment | | | | Number of Insured Patients who required Hospital Treatment | | | Number of Insured Patients who were sent back to their Panel Doctors | | | Approximate Percentage of Insured Patients to Total Number of Patients |
|---|---|---|---|---|---|---|---|---|---|---|---|
| Casualty | Medical | Surgical | Ophthal. | Total | Medical | Surgical | Ophtbal. | Medical | Surgical | Ophthal. | |
| 1913 ... | 534 | 4,071 | 41 | 4,846 | 189 | 2,383 | 41 | 345 | 1,683 | — | 18·0 |
| 1914 ... | 842 | 3.962 | 265 | 5,069 | 398 | 2,731 | 265 | 444 | 1,231 | — | 21·2 |
| 1915 ... | 660 | 3,975 | 207 | 4,842 | 259 | 2,729 | 207 | 401 | 1,247 | — | 25·0 |
| Out-Patients | Medical & Surgical | Ophthal. | Tuber-culous | Total | Medical & Surgical | Ophthal. | Tuber-culous | Medical & Surgical | Ophthal. | Tuber-culous | |
| 1913 ... | 4,135 | 583 | 408 | 5,126 | 3,503 | 583 | 408 | 632 | — | — | 19·2 |
| 1914 ... | 4,869 | 660 | 409 | 5.938 | 4,336 | 660 | 409 | 533 | — | — | 20·9 |
| 1915 ... | 4,379 | 634 | 461 | 5,474 | 3,803 | 634 | 461 | 576 | — | — | 21·1 |

